# Littlewood-Paley Operators on Morrey Spaces with Variable Exponent

**DOI:** 10.1155/2014/790671

**Published:** 2014-08-07

**Authors:** Shuangping Tao, Lijuan Wang

**Affiliations:** College of Mathematics and Statistics Science, Northwest Normal University, Lanzhou 730070, China

## Abstract

By applying the vector-valued inequalities for the Littlewood-Paley operators and their commutators on Lebesgue spaces with variable exponent, the boundedness of the Littlewood-Paley operators, including the Lusin area integrals, the Littlewood-Paley *g*-functions and *g*_μ_*-functions, and their commutators generated by BMO functions, is obtained on the Morrey spaces with variable exponent.

## 1. Introduction and Main Results

Let *ψ* ∈ *L* ^1^(*R* ^*n*^) and satisfy the following:

- ∫_*R*^*n*^_ *ψ*(*x*)*dx* = 0;
- |*ψ*(*x*)|⩽*C*(1 + |*x* | )^−*n*−*ɛ*^;
- |*ψ*(*x* + *y*) − *ψ*(*x*)|⩽*C* | *y*|^*γ*^(1 + |*x* | )^−*n*−*γ*−*ɛ*^, |*x* | ⩾2 | *y*|,

where *C*, *ɛ*, *γ* are all positive constants. Denote *ψ*_*t*_(*x*) = *t* ^−*n*^ *ψ*(*x*/*t*) with *t* > 0 and *x* ∈ *R* ^*n*^. Given a function *f* ∈ *L*_loc⁡_^1^(*R* ^*n*^), the Lusin area integral of *f* is defined by

(1) $$\begin{array}{c} S_{\psi ,a}\left(f\right)\left(x\right)={\left(\int_{\Gamma_{a}(x)}{\left|\psi_{t}*f\left(y\right)\right|}^{2}\frac{dy\;dt}{t^{n+1}}\right)}^{1/2}, \end{array}$$

where Γ_*a*_(*x*) denote the usual cone of aperture one

(2) $$\begin{array}{c} \Gamma_{a}\left(x\right)=\left\{\left(t,y\right)\in R_{+}^{n+1}:\left|y-x\right|<at,\;\;a\geq 0\right\}. \end{array}$$

As *a* = 1, we denote *S*_*ψ*,*a*_(*f*) as *S*_*ψ*_(*f*).

Now let us turn to the introduction of the other two Littlewood-Paley operators. It is well known that the Littlewood-Paley operators include also the Littlewood-Paley *g*-functions and the Littlewood-Paley *g*_μ_*-functions besides the Lusin area integrals. The Littlewood-Paley *g*-functions, which can be viewed as a “zero-aperture” version of *S*_*ψ*_, and *g*_μ_*-functions, which can be viewed as an “infinite-aperture” version of *S*_*ψ*_, are, respectively, defined by

(3) $$\begin{array}{c} g\left(f\right)\left(x\right)={\left(\overset{\infty }{\underset{0}{\int }}{\left|\psi_{t}*f\left(y\right)\right|}^{2}\frac{dt}{t}\right)}^{1/2}, \end{array}$$

(4) gμ∗(f)(x)=(∫R+n+1(tt+|x−y|)μn|ψt∗f(y)|2dy dttn+1)1/2,μ>0.

If we take *ψ* to be the Poisson kernel, then the functions defined above are the classical Littlewood-Paley operators.

Letting *b* ∈ *L*_loc⁡_^1^(*R* ^*n*^),  *m* ≥ 1, the corresponding *m*-order commutators of Littlewood-Paley operators above generated by a function *b* are defined by

(5) [bm,gψ](f)(x) =(∫0∞|∫Rn[b(x)−b(y)]mψt(x−y)f(y)dy|2dtt)1/2,

(6) [bm,Sψ,a](f)(x) =(∫Γa(x)|∫Rn[b(x)−b(z)]mψt(y−z)f(z)dz|2dy dttn+1)1/2,

(7) [bm,gμ∗](f)(x) =(∫R+n+1(tt+|x−y|)μn   ×|∫Rn[b(x)−b(z)]mψt(y−z)f(z)dz|2dy dttn+1)1/2,

where *μ* > 0.

The Littlewood-Paley operators are a class of important integral operators. Due to the fact that they play very important roles in harmonic analysis, PDE, and the other fields (see [1–3]), people pay much more attention to this class of operators. In 1995, Lu and Yang investigated the behavior of Littlewood-Paley operators in the space CBMO_*p*_  (*R* ^*n*^) in [4]. In 2005, Zhang and Liu proved the commutator [*b*, *g*_*ψ*_] is bounded on *L* ^*p*^(*ω*) in [5]. In 2009, Xue and Ding gave the weighted estimate for Littlewood-Paley operators and their commutators (see [6]). There are some other results about Littlewood-Paley operators in [7–9] and so forth.

On the other hand, Lebesgue spaces with variable exponent *L* ^*p*(·)^(*R* ^*n*^) become one class of important research subject in analysis filed due to the fundamental paper [10] by Kováčik and Rákosník. In the past twenty years, the theory of these spaces has made progress rapidly, and the study of which has many applications in fluid dynamics, elasticity, calculus of variations, and differential equations with nonstandard growth conditions (see [11–15]). In [16], Cruz-Uribe et al. stated that the extrapolation theorem leads the boundedness of some classical operators including the commutator on *L* ^*p*(·)^(*R* ^*n*^). Karlovich and Lerner also independently obtained the boundedness of the singular integrals commutator on Lebesgue spaces with variable exponent in [17]. In 2009 and 2010, Izuki considered the boundedness of vector-valued sublinear operators and fractional integrals on Herz-Morrey spaces with variable exponent in [18, 19], respectively. In 2013, Ho in [20] introduced a class of Morrey spaces with variable exponent *M*_*p*(·),*u*_ and studied the boundedness of the fractional integral operators on *M*_*p*(·),*u*_.

Inspired by the results mentioned previously, in this paper we will consider the vector-valued inequalities of the Littlewood-Paley operators and their *m*-order commutators on Morrey spaces with variable exponent. Before stating our main results, we need to recall some relevant definitions and notations.

Let *E* be a Lebesgue measurable set in *R* ^*n*^ with measure |*E* | >0.

**Definition 1 (see [10]).**

Let *p*(·) : *E* → [1, *∞*) be a measurable function.

The Lebesgue space with variable exponent *L* ^*p*(·)^(*E*) is defined by

(8) Lp(·)(E)={f  is  measurable:  ∫E(|f(x)|η)p(x)dx<∞for  some  constant  η>0}.

The space *L*_loc⁡_^*p*(·)^(*E*) is defined by

(9) Lloc⁡p(·)(E) ={f  is  measurable:  f∈Lp(·)(K)for  all  compact  subsets  K⊂E}.

The Lebesgue space *L* ^*p*(·)^(*E*) is a Banach space with the norm defined by

(10) $$\begin{array}{c} {||f||}_{L^{p(\cdot )}(E)}=\inf\left\{\eta >0:\int_{E}{\left(\frac{\left|f\left(x\right)\right|}{\eta }\right)}^{p(x)}dx\leq 1\right\}. \end{array}$$

**Remark 2 .**

(1) Note that if the function *p*(*x*) = *p*_0_ is a constant function, then *L* ^*p*(·)^(*R* ^*n*^) equals *L* ^*p*_0_^(*R* ^*n*^). This implies that the Lebesgue spaces with variable exponent generalize the usual Lebesgue spaces. And they have many properties in common with the usual Lebesgue spaces.

(2) Denote *p*_−_ : = ess inf{*p*(*x*) : *x* ∈ *E*},  *p*_+_ : = ess sup{*p*(*x*) : *x* ∈ *E*}. Then *P*(*E*) consists of all *p*(·) satisfying *p*_−_ > 1 and *p*_+_ < *∞*.

(3) The Hardy-Littlewood maximal operator *M* is defined by

(11) $$\begin{array}{c} M\left(f\right)\left(x\right)=\underset{Q∋x}{\sup}\frac{1}{\left|Q\right|}\int_{Q}\left|f\left(y\right)\right|dy. \end{array}$$

Denote *B*(*E*) to be the set of all functions *p*(·) ∈ *P*(*E*) satisfying the condition that *M* is bounded on *L* ^*p*(·)^(*E*).

(4) Let *p*(·) ∈ *B*(*R* ^*n*^). Denote *κ*_*p*(·)_ = sup⁡{*q* > 1 : *p*(·)/*q* ∈ *B*(*R* ^*n*^)} and *e*_*p*(·)_ is the conjugate exponent of *κ*_*p*′(·)_ (see [20]).

**Definition 3 (see [20]).**

Let *p*(*x*) ∈ *L* ^*∞*^(*R* ^*n*^), 1 < *p*(*x*) < *∞*. If there exists a constant *C* > 0 such that, for any *x* ∈ *R* ^*n*^ and *r* > 0, Lebesgue measurable function *u*(*x*, *r*) : *R* ^*n*^ × (0, *∞*)→(0, *∞*) satisfying

(12) $$\begin{array}{c} \overset{\infty }{\underset{j=0}{\sum }}\frac{{||\chi_{B(x,r)}||}_{L^{p(\cdot )}(R^{n})}}{{||\chi_{B(x,2^{j+1}r)}||}_{L^{p(\cdot )}(R^{n})}}u\left(x,2^{j+1}r\right)\leq Cu\left(x,r\right), \end{array}$$

then one says *u* is a Morrey weight function for *L* ^*p*(·)^(*R* ^*n*^). One denotes the class of Morrey weight functions by *W*_*p*(·)_.

**Definition 4 (see [20]).**

Let *p*(*x*) ∈ *B*(*R* ^*n*^), *u*(*x*, *r*) ∈ *W*_*p*(·)_. Then the Morrey spaces with variable exponent *M*_*p*(·),*u*_(*R* ^*n*^) are defined by

(13) $$\begin{array}{c} M_{p(\cdot ),u}\left(R^{n}\right)=\left\{f\;\text{is}\;\;\text{measurable:}\;\;{||f||}_{M_{p(\cdot ),u}(R^{n})}<\infty \right\}, \end{array}$$

where

(14) $$\begin{array}{c} {||f||}_{M_{p(\cdot ),u}(R^{n})}=\underset{x\in R^{n},R>0}{\sup}\frac{1}{u\left(x,R\right)}{||f\chi_{B(x,R)}||}_{L^{p(\cdot )}(R^{n})}. \end{array}$$

**Remark 5 .**

(1) If *u*(*x*, *r*) ≡ 1, then *M*_*p*(·),*u*_(*R* ^*n*^) is the Lebesgue spaces with variable exponent *L* ^*p*(·)^(*R* ^*n*^).

(2) Notice that if *p*(*x*) ≡ *p*, 1 < *p* < *∞*, is a constant function, then formula (12) can be rewritten as an integral in form. To be precise, formula (12) can be rewritten in the following form (see [20]):

(15) $$\begin{array}{c} \overset{\infty }{\underset{r}{\int }}\frac{u\left(x,t\right)}{t^{n/p+1}}dt\leq C\frac{u\left(x,r\right)}{r^{n/p}},\;r>0,\;\;\forall x\in R^{n}. \end{array}$$

Let 0 < *α* < *n*. By the the conditions of Morrey weight functions mentioned in [21]

(16) $$\begin{array}{c} \overset{\infty }{\underset{r}{\int }}\frac{u^{p}\left(x,t\right)}{t^{n-\alpha /p+1}}dt\leq C\frac{u^{p}\left(x,r\right)}{r^{n-\alpha /p}},\;r>0,\;\;\forall x\in R^{n} \end{array}$$

and Hölder's inequality, via simple calculation, we have

(17) ∫r∞u(x,t)tn/p+1dt  ≤C{∫r∞up(x,t)tn−α/p+1dt}1/p{∫r∞1tαp′+1dt}1/p′  ≤Cu(x,r)rn/p.

From this, it follows that if *p*(*x*) ≡ *p*, 1 < *p* < *∞*, is a constant function, then condition (12) is weaker than condition (16). Thus, the class of the Morrey spaces introduced in Definition 4 is more wide than that satisfying condition (1.8) in [21]. More studies of common Morrey spaces can be seen in [22, 23] and so forth.

(3) If *u*(*x*, *r*) = |*B*(*x*, *r*)|^1/*p*(*x*)−1/*q*(*x*)^,  *p*(*x*) ≤ *q*(*x*), then the space mentioned in Definition 4 is the Morrey space with variable exponent introduced in [24]. And when 1 < *s* < *κ*_*p*′(*x*)_,  1/*p*(*x*) − 1/*q*(*x*) < 1 − 1/*s*, it is easy to see *u*(*x*, *r*) satisfying condition (12). That is because it follows from *p*(*x*) ∈ *B*(*R* ^*n*^),  1 < *s* < *κ*_*p*′(*x*)_, that (see [20])

(18) $$\begin{array}{c} \frac{{||\chi_{B(x,r)}||}_{L^{p(\cdot )}(R^{n})}}{{||\chi_{B(x,2^{j+1}r)}||}_{L^{p(\cdot )}(R^{n})}}\leq C2^{jn(1/s-1)},\;\forall x\in R^{n},\;\;r>0,\;\;j\in N. \end{array}$$

For Littlewood-Paley operators *S*_*ψ*,*a*_, *g*_*ψ*_, and *g*_μ_*, in this paper, we have the following results.

**Theorem 6 .**

Suppose that function *ψ* ∈ *L* ^1^(*R* ^*n*^) satisfies (i)–(iii) and *S*_*ψ*,*a*_ is defined by (1). If *u* ∈ *W*_*p*(·)_, *p*(·) ∈ *B*(*R* ^*n*^), 1 < *r* < *∞*, then there exists a constant *C* > 0 independent of *f* such that, for any function sequences {*f*_*h*_}_*h*=1_^*∞*^ with ||{∑_*h*_|*f*_*h*_|^*r*^}^1/*r*^||_*M*_*p*(·),*u*_(*R*^*n*^)_ < *∞*, the following inequality holds:

(19) $$\begin{array}{c} {||{\left\{\underset{h}{\sum }{\left|S_{\psi ,a}\left(f_{h}\right)\right|}^{r}\right\}}^{1/r}||}_{M_{p(\cdot ),u}(R^{n})}\leq C{||{\left\{\underset{h}{\sum }{\left|f_{h}\right|}^{r}\right\}}^{1/r}||}_{M_{p(\cdot ),u}(R^{n})}. \end{array}$$

**Theorem 7 .**

Suppose that *g*_*ψ*_ is defined by (3). Then under the same condition as the one in Theorem 6, there exists a constant *C* > 0 independent of *f* such that, for any function sequences {*f*_*h*_}_*h*=1_^*∞*^ with ||{∑_*h*_|*f*_*h*_|^*r*^}^1/*r*^||_*M*_*p*(·),*u*_(*R*^*n*^)_ < *∞*, the following inequality holds:

(20) $$\begin{array}{c} {||{\left\{\underset{h}{\sum }{\left|g_{\psi }\left(f_{h}\right)\right|}^{r}\right\}}^{1/r}||}_{M_{p(\cdot ),u}(R^{n})}\leq C{||{\left\{\underset{h}{\sum }{\left|f_{h}\right|}^{r}\right\}}^{1/r}||}_{M_{p(\cdot ),u}(R^{n})}. \end{array}$$

**Theorem 8 .**

Suppose that *g*_μ_* is defined by (4) and *μ* > 3 + 2(*ɛ* + *γ*)/*n*,  0 < *γ* < *ɛ*. Then under the same condition as the one in Theorem 6, there exists a constant *C* > 0 independent of *f* such that, for any function sequences {*f*_*h*_}_*h*=1_^*∞*^ with ||{∑_*h*_|*f*_*h*_|^*r*^}^1/*r*^||_*M*_*p*(·),*u*_(*R*^*n*^)_ < *∞*, the following inequality holds:

(21) $$\begin{array}{c} {||{\left\{\underset{h}{\sum }{\left|g_{\mu }^{*}\left(f_{h}\right)\right|}^{r}\right\}}^{1/r}||}_{M_{p(\cdot ),u}(R^{n})}\leq C{||{\left\{\underset{h}{\sum }{\left|f_{h}\right|}^{r}\right\}}^{1/r}||}_{M_{p(\cdot ),u}(R^{n})}. \end{array}$$

For commutators [*b* ^*m*^, *S*_*ψ*,*a*_],  [*b* ^*m*^, *g*_*ψ*_], and [*b* ^*m*^, *g*_μ_*], we have the following results.

**Theorem 9 .**

Suppose that function *ψ* ∈ *L* ^1^(*R* ^*n*^) satisfies (i)–(iii) and [*b* ^*m*^, *S*_*ψ*,*a*_] is defined by (5). Let *b* ∈ *BMO*(*R* ^*n*^), *p*(·) ∈ *B*(*R* ^*n*^), *m* ≥ 1,  1 < *r* < *∞*. If, for any *x* ∈ *R* ^*n*^ and *r*_0_ > 0, function *u* satisfies

(22) $$\begin{array}{c} \overset{\infty }{\underset{j=0}{\sum }}\frac{{||\chi_{B(x,r_{0})}||}_{L^{p(\cdot )}(R^{n})}}{{||\chi_{B(x,2^{j+1}r_{0})}||}_{L^{p(\cdot )}(R^{n})}}j^{m}u\left(x,2^{j+1}r_{0}\right)\leq Cu\left(x,r_{0}\right), \end{array}$$

then there exists a constant *C* > 0 independent of *f* such that, for any function sequences {*f*_*h*_}_*h*=1_^*∞*^ with ||{∑_*h*_|*f*_*h*_|^*r*^}^1/*r*^||_*M*_*p*(·),*u*_(*R*^*n*^)_ < *∞*, the following inequality holds:

(23) ||{∑h|[bm,Sψ,a](fh)|r}1/r||Mp(·),u(Rn) ≤C||{∑h|fh|r}1/r||Mp(·),u(Rn).

**Theorem 10 .**

Suppose that [*b* ^*m*^, *g*_*ψ*_] is defined by (6). Then under the same condition as the one in Theorem 9, there exists a constant *C* > 0 independent of *f* such that, for any function sequences {*f*_*h*_}_*h*=1_^*∞*^ with ||{∑_*h*_|*f*_*h*_|^*r*^}^1/*r*^||_*M*_*p*(·),*u*_(*R*^*n*^)_ < *∞*, the following inequality holds:

(24) ||{∑h|[bm,gψ](fh)|r}1/r||Mp(·),u(Rn) ≤C||{∑h|fh|r}1/r||Mp(·),u(Rn).

**Theorem 11 .**

Suppose that [*b* ^*m*^, *g*_μ_*] is defined by (7), *μ* > 3 + 2(*ɛ* + *γ*)/*n*, and 0 < *γ* < *ɛ*. Then under the same condition as the one in Theorem 9, there exists a constant *C* > 0 independent of *f* such that, for any function sequences {*f*_*h*_}_*h*=1_^*∞*^ with ||{∑_*h*_|*f*_*h*_|^*r*^}^1/*r*^||_*M*_*p*(·),*u*_(*R*^*n*^)_ < *∞*, the following inequality holds:

(25) ||{∑h|[bm,gμ∗](fh)|r}1/r||Mp(·),u(Rn) ≤C||{∑h|fh|r}1/r||Mp(·),u(Rn).

**Remark 12 .**

(1) It is easy to see that condition (22) in Theorem 9 is stronger than condition (12) in Definition 3. Therefore, if a function *u* satisfies condition (22), then *u* ∈ *W*_*p*(·)_.

(2) The function *u* which satisfies (22) exists. In fact, if we take *τ* : 0 ≤ *τ* < 1/*e*_*p*(·)_ such that function *u* satisfies

(26) $$\begin{array}{c} \frac{u\left(x,2r\right)}{u\left(x,r\right)}\leq 2^{n\tau },\;\forall x\in R^{n},\;\;r>0, \end{array}$$

then *u* ∈ *W*_*p*(·)_. That is because, for any *τ* < 1/*e*_*p*(·)_, there exists *s* < *κ*_*p*′(·)_ such that *τ* < 1 − 1/*s* < 1 − 1/*κ*_*p*′(·)_ = 1/*e*_*p*(·)_. Hence, it follows from (18) that

(27) ∑j=0∞||χB(x,r)||Lp(·)(Rn)||χB(x,2j+1r)||Lp(·)(Rn)jmu(x,2j+1r) ≤C∑j=0∞jm2−jn(1−1/s)2jnτu(x,r)≤Cu(x,r).

We end this section by introducing some conventional notations which will be used later. Throughout this paper, given a function *f*, we denote the mean value of *f* on *E* by *f*_*E*_ = :(1/|*E*|)∫_*E*_ *f*(*x*)*dx*. *p*′(·) means the conjugate exponent of *p*(·); namely, 1/*p*(*x*) + 1/*p*′(*x*) = 1 holds. *C* always means a positive constant independent of the main parameters and may change from one occurrence to another.

## 2. Preliminary Lemmas 

In this section, we introduce some conclusions which will be used in the proofs of our main results.

**Lemma 13 (see [10] (generalized Hölder's inequality)).**

Let *p*(·), *p*_1_(·), *p*_2_(·) ∈ *P*(*R* ^*n*^).

(1) For any *f* ∈ *L* ^*p*(·)^(*R* ^*n*^), *g* ∈ *L* ^*p*′(·)^(*R* ^*n*^),
  (28) $$\begin{array}{c} \int_{R^{n}}\left|f\left(x\right)g\left(x\right)\right|dx\leq C_{p}{||f||}_{L^{p(\cdot )}(R^{n})}{||g||}_{L^{p^{\prime }(\cdot )}(R^{n})}, \end{array}$$
 where *C*_*p*_ = 1 + 1/*p*_−_ − 1/*p*_+_.
(2) For any *f* ∈ *L* ^*p*_1_(·)^(*R* ^*n*^), *g* ∈ *L* ^*p*_2_(·)^(*R* ^*n*^), when 1/*p*(*x*) = 1/*p*_1_(*x*) + 1/*p*_2_(*x*), one has
  (29) $$\begin{array}{c} {||f\left(x\right)g\left(x\right)||}_{L^{p(\cdot )}(R^{n})}\leq C_{p_{1},p_{2}}{||f||}_{L^{p_{1}(\cdot )}(R^{n})}{||g||}_{L^{p_{2}(\cdot )}(R^{n})}, \end{array}$$
 where *C*_*p*_1_,*p*_2__ = (1 + 1/*p*_1_−__ − 1/*p*_1_+__)^1/*p*_−_^.

**Lemma 14 (see [17]).**

If *p*(·) ∈ *B*(*R* ^*n*^), then there exist constants *δ*_1_, *δ*_2_, *C* > 0, such that, for all balls *B* ⊂ *R* ^*n*^ and all measurable subsets *S* ⊂ *B*,

(30) $$\begin{array}{c} \frac{{||\chi_{B}||}_{L^{p(\cdot )}(R^{n})}}{{||\chi_{S}||}_{L^{p(\cdot )}(R^{n})}}\leq C\frac{\left|B\right|}{\left|S\right|},\\ \frac{{||\chi_{S}||}_{L^{p^{\prime }(\cdot )}(R^{n})}}{{||\chi_{B}||}_{L^{p^{\prime }(\cdot )}(R^{n})}}\leq C{\left(\frac{\left|S\right|}{\left|B\right|}\right)}^{\delta_{1}},\\ \frac{{||\chi_{S}||}_{L^{p(\cdot )}(R^{n})}}{{||\chi_{B}||}_{L^{p(\cdot )}(R^{n})}}\leq C{\left(\frac{\left|S\right|}{\left|B\right|}\right)}^{\delta_{2}}. \end{array}$$

**Remark 15 .**

From formula (12), it follows that

(31) $$\begin{array}{c} \frac{{||\chi_{B(x,r)}||}_{L^{p(\cdot )}(R^{n})}}{{||\chi_{B(x,2^{j+1}r)}||}_{L^{p(\cdot )}(R^{n})}}u\left(x,2^{j+1}r\right)\leq Cu\left(x,r\right),\;\forall j\in N. \end{array}$$

Thus, by Lemma 14, we have, ∀*j* ∈ *N*,

(32) $$\begin{array}{c} \frac{u\left(x,2^{j}r\right)}{u\left(x,r\right)}\leq C\frac{{||\chi_{B(x,2^{j}r)}||}_{L^{p(\cdot )}(R^{n})}}{{||\chi_{B(x,r)}||}_{L^{p(\cdot )}(R^{n})}}\leq C\frac{\left|B\left(x,2^{j}r\right)\right|}{\left|B\left(x,r\right)\right|}\leq C2^{nj}. \end{array}$$

**Lemma 16 (see [18]).**

If *p*(·) ∈ *B*(*R* ^*n*^), then there exists constant *C* > 0, such that, for all balls *B* ⊂ *R* ^*n*^,

(33) $$\begin{array}{c} \frac{1}{\left|B\right|}{||\chi_{B}||}_{L^{p(\cdot )}(R^{n})}{||\chi_{B}||}_{L^{p^{\prime }(\cdot )}(R^{n})}\leq C. \end{array}$$

**Lemma 17 (see [25]).**

Let *b* ∈ *BMO*(*R* ^*n*^);  *m* is a positive integer. There exist constants *C* > 0, such that, for any *k*, *j* ∈ *Z* with *k* > *j*,

1. *C* ^−1^||*b*||_∗_^*m*^ ≤ sup⁡_*B*_(1/||*χ*_*B*_||_*L*^*p*(·)^(*R*^*n*^)_)||(*b*−*b*_*B*_)^*m*^ *χ*_*B*_||_*L*^*p*(·)^(*R*^*n*^)_ ≤ *C*||*b*||_∗_^*m*^;
2. ||(*b*−*b*_*B*_*j*__)^*m*^ *χ*_*B*_*k*__||_*L*^*p*(·)^(*R*^*n*^)_ ≤ *C*(*k* − *j*)^*m*^||*b*||_∗_^*m*^||*χ*_*B*_*k*__||_*L*^*p*(·)^(*R*^*n*^)_.

**Lemma 18 (see [26]).**

Let *ψ* ∈ *L* ^1^(*R* ^*n*^) satisfy (i)–(iii). If *p*(·) ∈ *B*(*R* ^*n*^),  1 < *q* < *∞*, then for all bounded compactly support functions *f*_*j*_ such that {*f*_*j*_}_*j*=1_^*∞*^ ∈ *L* ^*p*(·)^(*l* ^*q*^), that is, ||(∑_*j*_(|*f*_*j*_|)^*q*^)^1/*q*^||_*L*^*p*(·)^(*R*^*n*^)_ < *∞*, the following vector-valued inequalities hold:

1. ||(∑_*j*_|*S*_*ψ*,*a*_(*f*_*j*_)|^*q*^)^1/*q*^||_*L*^*p*(·)^(*R*^*n*^)_ ≤ *C*||(∑_*j*_|*f*_*j*_|^*q*^)^1/*q*^||_*L*^*p*(·)^(*R*^*n*^)_,
2. ||(∑_*j*_|*g*_*ψ*_(*f*_*j*_)|^*q*^)^1/*q*^||_*L*^*p*(·)^(*R*^*n*^)_ ≤ *C*||(∑_*j*_|*f*_*j*_|^*q*^)^1/*q*^||_*L*^*p*(·)^(*R*^*n*^)_,
3. ||(∑_*j*_|*g*_μ_*(*f*_*j*_)|^*q*^)^1/*q*^||_*L*^*p*(·)^(*R*^*n*^)_ ≤ *C*||(∑_*j*_|*f*_*j*_|^*q*^)^1/*q*^||_*L*^*p*(·)^(*R*^*n*^)_,

where *μ* > 2, 0 < *γ* < min⁡{(*μ* − 2)*n*/2, *ɛ*}.

**Lemma 19 (see [26]).**

Let *b* ∈ *BMO*, *ψ* ∈ *L* ^1^(*R* ^*n*^) satisfy (i)–(iii), *m* ∈ *N*∖{0}. If *p*(·) ∈ *B*(*R* ^*n*^), 1 < *q* < *∞*, then for all bounded compactly support functions *f*_*j*_ such that {*f*_*j*_}_*j*=1_^*∞*^ ∈ *L* ^*p*(·)^(*l* ^*q*^), that is, ||(∑_*j*_(|*f*_*j*_|)^*q*^)^1/*q*^||_*L*^*p*(·)^(*R*^*n*^)_ < *∞*, the following vector-valued inequalities hold:

1. ||(∑_*j*_|[*b* ^*m*^,*S*_*ψ*,*a*_](*f*_*j*_)|^*q*^)^1/*q*^||_*L*^*p*(·)^(*R*^*n*^)_ ≤ *C*||(∑_*j*_|*f*_*j*_|^*q*^)^1/*q*^||_*L*^*p*(·)^(*R*^*n*^)_,
2. ||(∑_*j*_|[*b* ^*m*^,*g*_*ψ*_](*f*_*j*_)|^*q*^)^1/*q*^||_*L*^*p*(·)^(*R*^*n*^)_ ≤ *C*||(∑_*j*_|*f*_*j*_|^*q*^)^1/*q*^||_*L*^*p*(·)^(*R*^*n*^)_,
3. ||(∑_*j*_|[*b* ^*m*^,*g*_μ_*](*f*_*j*_)|^*q*^)^1/*q*^||_*L*^*p*(·)^(*R*^*n*^)_ ≤ *C*||(∑_*j*_|*f*_*j*_|^*q*^)^1/*q*^||_*L*^*p*(·)^(*R*^*n*^)_,

where *μ* > 2,  0 < *γ* < min⁡{(*μ* − 2)*n*/2, *ɛ*}.

## 3. Proofs of Main Results 

Next, let us show the proofs of Theorems 6–11, respectively.

**Proof of Theorem 6.**

Let ||{*f*_*h*_}_*h*_||_*l*^*r*^_ ∈ *M*_*p*(·),*u*_(*R* ^*n*^); for any *x*_0_ ∈ *R* ^*n*^, *r*_0_ > 0, denote

(34) $$\begin{array}{c} f_{h}\left(x\right)≜f_{h}^{0}\left(x\right)+\overset{\infty }{\underset{j=1}{\sum }}f_{h}^{j}\left(x\right), \end{array}$$

where *f*_*h*_^0^ = *f*_*h*_ *χ*_*B*(*x*_0_,2*r*_0_)_,  *f*_*h*_^*j*^ = *f*_*h*_ *χ*_*B*(*x*_0_,2^*j*+1^*r*_0_)∖*B*(*x*_0_,2^*j*^*r*_0_)_, *j* ∈ *N*∖{0}.

Noting that, in order to prove Theorem 6, it is enough to show that the following inequality holds:

(35) 1u(x0,r0)||{∑h|Sψ,a(fh)|r}1/rχB(x0,r0)||Lp(·)(Rn) ≤C||{∑h|fh|r}1/r||Mp(·),u(Rn).

Thus,

(36) 1u(x0,r0)||{∑h|Sψ,a(fh)|r}1/rχB(x0,r0)||Lp(·)(Rn) ≤Cu(x0,r0)||{∑h|Sψ,a(fh0)|r}1/rχB(x0,r0)||Lp(·)(Rn)  +Cu(x0,r0)∑j=1∞||{∑h|Sψ,a(fhj)|r}1/rχB(x0,r0)||Lp(·)(Rn) ≜D1+D2.

For the term *D*_1_, notice that supp *f*_*h*_^0^ ⊂ *B*(*x*_0_, 2*r*_0_); using Lemma 18 and (32), it is easy to see that

(37) D1≤Cu(x0,r0)||{∑h|fh0|r}1/r||Lp(·)(Rn)≤Cu(x0,2r0)u(x0,r0)1u(x0,2r0) ×||{∑h|fh|r}1/rχB(x0,2r0)||Lp(·)(Rn)≤C2nsup⁡y∈Rn,R>01u(y,R) ×||{∑h|fh|r}1/rχB(y,R)||Lp(·)(Rn)≤C||{∑h|fh|r}1/r||Mp(·),u(Rn).

We now turn to estimate *D*_2_. To do this, we need to consider *S*_*ψ*,*a*_ first. Without loss of generality, we may assume that *a* ≥ 1. Let

(38) $$\begin{array}{c} \Gamma^{\prime }=\left\{y\in R^{n}:\left|y-x\right|<at,\;\;y\leq 2^{j+1}r_{0}\right\},\\ \Gamma^{\prime \prime }=\left\{y\in R^{n}:\left|y-x\right|<at,\;\;y>2^{j+1}r_{0}\right\}. \end{array}$$

Then, by Minkowski's inequality, we have

(39) Sψ,a(fhj)(x) =(∫Γa(x)|∫Rnt−nψ(y−zt)fhj(z)dz|2dy dttn+1)1/2 ≤∫Rn|fhj(z)|(∫Γa(x)t−3n−1|ψ(y−zt)|2dy dt)1/2dz ≤∫Rn|fhj(z)|(∫0∞∫Γ′t−3n−1|ψ(y−zt)|2dy dt)1/2dz  +∫Rn|fhj(z)|(∫0∞∫Γ′′t−3n−1|ψ(y−zt)|2dy dt)1/2dz.

Observe that if *x* ∈ *B*(*x*_0_, *r*_0_),  *z* ∈ *B*(*x*_0_, 2^*j*+1^ *r*_0_)∖*B*(*x*_0_, 2^*j*^ *r*_0_),  *j* ≥ 1, then

(40) t+|y−z|≥|x−y|a+|y−z|≥|x−y|+|y−z|a≥|z|−|x|a≥|z|2a.

Therefore, it follows from condition (ii) that

(41) ∫0∞∫Γ′t−3n−1|ψ(y−zt)|2dy dt ≤∫0∞∫Γ′t−3n−1(1+|y−z|t)−2(n+ɛ)dy dt ≤∫02j+1r0∫Γ′t2ɛ−n−1(t+|y−z|)2(n+ɛ)dy dt  +∫2j+1r0∞∫Γ′t2ɛ−n−1(t+|y−z|)2(n+ɛ)dy dt ≤Ca2(n+ɛ)|z|2(n+ɛ)∫02j+1r0∫|x−y|<att2ɛ−n−1dy dt  +Ca2n|z|2n∫2j+1r0∞∫|x−y|<att−3n−1dy dt ≤Ca2(n+ɛ)|z|2(n+ɛ)∫02j+1r0ant2ɛ−1dy dt  +C∫2j+1r0∞ant−2n−1dy dt ≤Ca3n+2ɛ(2jr0)−2n.

On the other hand, we denote

(42) ∫0∞∫Γ′′t−3n−1|ψ(y−zt)|2dy dt ≤C∫0∞∫Γ′′t−3n−1|ψ(y−zt)−ψ(yt)|2dy dt  +C∫0∞∫Γ′′t−3n−1|ψ(yt)|2dy dt ≜I+II.

Note that if *y* > 2^*j*+1^ *r*_0_, then *t* > |*x* − *y*|/*a* ≥ (|*x*| − |*y*|)/*a* > (2^*j*+1^ *r*_0_ − *r*_0_)/*a* ≥ 2^*j*^ *r*_0_/*a*. Thus, by condition (iii), we get

(43) I≤C∫2jr0/a∞∫Γ′′t−3n−1(|z|t)2γ(1+|y|t)−2(n+γ+ɛ)dy dt≤C(2jr0)2γ∫2jr0/a∞∫|x−y|<att−3n−2γ−1dy dt≤C(2jr0)2γan∫2jr0/a∞t−2n−2γ−1dy dt≤Ca3n+2γ(2jr0)−2n.

And by condition (ii), similar to the estimate of *I*, we obtain

(44) II≤C∫2jr0/a∞∫Γ′′t−3n−1(|z|t)2γ(1+|y|t)−2(n+ɛ)dy dt≤C∫2jr0/a∞∫Γ′′t2ɛ−n−1(t+|y|)2(n+ɛ)dy dt≤Ca3n(2jr0)−2n.

Hence, from the estimates above, it follows that

(45) $$\begin{array}{c} S_{\psi ,a}\left(f_{h}^{j}\right)\left(x\right)\leq Ca^{3n/2+\gamma +ɛ}{\left(2^{j}r_{0}\right)}^{-n}{||f_{h}^{j}||}_{L^{1}(R^{n})}. \end{array}$$

Thus,

(46) D2≤Cu(x0,r0)∑j=1∞||{∑h|a3n/2+γ+ɛ(2jr0)−n×||fhj||L1(Rn)|r}1/rχB(x0,r0)||Lp(·)(Rn)≤a3n/2+γ+ɛCu(x0,r0)∑j=1∞(2jr0)−n||χB(x0,r0)||Lp(·)(Rn) ×||{∑h|fhj|r}1/r||L1(Rn).

Therefore, applying the generalized Hölder's inequality, Lemma 16, and (12), we have

(47) D2≤a3n/2+γ+ɛCu(x0,r0)∑j=1∞(2jr0)−n||χB(x0,r0)||Lp(·)(Rn) ×||{∑h|fhj|r}1/r||Lp(·)(Rn)||χB(x0,2j+1r0)||Lp′(·)(Rn)≤Cu(x0,r0)∑j=1∞(2jr0)−n|B(x0,2j+1r0)| ×||χB(x0,r0)||Lp(·)(Rn)||χB(x0,2j+1r0)||Lp(·)(Rn)×||{∑h|fhj|r}1/r||Lp(·)(Rn)≤Cu(x0,r0)∑j=1∞||χB(x0,r0)||Lp(·)(Rn)||χB(x0,2j+1r0)||Lp(·)(Rn)u(x0,2j+1r0) ×1u(x0,2j+1r0)||{∑h|fh|r}1/rχB(x0,2j+1r0)||Lp(·)(Rn)≤Csup⁡y∈Rn,R>01u(y,R)||{∑h|fh|r}1/rχB(y,R)||Lp(·)(Rn)≤C||{∑h|fh|r}1/r||Mp(·),u(Rn).

Adding up the estimates of *D*_1_, *D*_2_, we obtain

(48) 1u(x0,r0)||{∑h|Sψ,a(fh)|r}1/rχB(x0,r0)||Lp(·)(Rn) ≤C||{∑h|fh|r}1/r||Mp(·),u(Rn).

This completes the proof of Theorem 6.

Now let us prove Theorems 7 and 8 in brief.

**Proof of Theorem 7.**

For *g*_*ψ*_, similar to the estimate of *S*_*ψ*,*a*_, via a simple calculation, we get that (see [26]) if *x* ∈ *B*(*x*_0_, *r*_0_), supp *f*_*h*_^*j*^ ⊂ *B*(*x*_0_, 2^*j*+1^ *r*_0_)∖*B*(*x*_0_, 2^*j*^ *r*_0_),  *j* ≥ 1, then

(49) $$\begin{array}{c} g_{\psi }\left(f_{h}^{j}\right)\left(x\right)\leq C{\left(2^{j}r_{0}\right)}^{-n}{||f_{h}^{j}||}_{L^{1}(R^{n})}. \end{array}$$

Hence, similar to the proof of Theorem 6, it follows from inequality (2) in Lemma 18 that

(50) $$\begin{array}{c} {||{\left\{\underset{h}{\sum }{\left|g_{\psi }\left(f_{h}\right)\right|}^{r}\right\}}^{1/r}||}_{M_{p(\cdot ),u}(R^{n})}\leq C{||{\left\{\underset{h}{\sum }{\left|f_{h}\right|}^{r}\right\}}^{1/r}||}_{M_{p(\cdot ),u}(R^{n})}. \end{array}$$

This accomplishes the proof of Theorem 7.

**Proof of Theorem 8.**

For *g*_μ_*, by the definitions of *S*_*ψ*,*a*_ and *g*_μ_*, we have

(51) gμ∗(f)(x) =(∫0∞∫Rn(tt+|x−y|)μn|ψt∗f(y)|2dy dttn+1)1/2 ≤(∫0∞∫|x−y|<t(tt+|x−y|)μn|ψt∗f(y)|2dy dttn+1)1/2  +∑l=1∞(∫0∞∫2l−1t≤|x−y|<2lt(tt+|x−y|)μn‍            ×|ψt∗f(y)|2dy dttn+1)1/2 ≤Sψ,a(fhj)(x)+∑l=1∞(1+2l−1)−μn/2Sψ,2la(fhj)(x).

According to the estimate of *S*_*ψ*,*a*_ in the proof of Theorem 6, we know that if *x* ∈ *B*(*x*_0_, *r*_0_), supp *f*_*h*_^*j*^ ⊂ *B*(*x*_0_, 2^*j*+1^ *r*_0_)∖*B*(*x*_0_, 2^*j*^ *r*_0_), *j* ≥ 1, then

(52) $$\begin{array}{c} S_{\psi ,a}\left(f_{h}^{j}\right)\left(x\right)\leq Ca^{3n/2+ɛ+\gamma }{\left(2^{j}r_{0}\right)}^{-n}{||f_{h}^{j}||}_{L^{1}(R^{n})}. \end{array}$$

Thus, as *μ* > 3 + 2(*ɛ* + *γ*)/*n*, we obtain

(53) gμ∗(fhj)(x)≤Ca3n/2+ɛ+γ(1+∑l=1∞2(3n/2+ɛ+γ−μn/2)) ×(2jr0)−n||fhj||L1(Rn)≤Ca3n/2+ɛ+γ(2jr0)−n||fhj||L1(Rn).

Hence, also similar to the proof of Theorem 6, and from inequality (3) in Lemma 18, it follows that

(54) $$\begin{array}{c} {||{\left\{\underset{h}{\sum }{\left|g_{\mu }^{*}\left(f_{h}\right)\right|}^{r}\right\}}^{1/r}||}_{M_{p(\cdot ),u}(R^{n})}\leq C{||{\left\{\underset{h}{\sum }{\left|f_{h}\right|}^{r}\right\}}^{1/r}||}_{M_{p(\cdot ),u}(R^{n})}. \end{array}$$

This finishes the proof of Theorem 8.

**Proof of Theorem 9.**

Let *b* ∈ BMO, ||{*f*_*h*_}_*h*_||_*l*^*r*^_ ∈ *M*_*p*(·),*u*_(*R* ^*n*^). For any *x*_0_ ∈ *R* ^*n*^, *r*_0_ > 0, denote

(55) $$\begin{array}{c} f_{h}\left(x\right)≜f_{h}^{0}\left(x\right)+\overset{\infty }{\underset{j=1}{\sum }}f_{h}^{j}\left(x\right), \end{array}$$

where *f*_*h*_^0^ = *f*_*h*_ *χ*_*B*(*x*_0_,2*r*_0_)_ and *f*_*h*_^*j*^ = *f*_*h*_ *χ*_*B*(*x*_0_,2^*j*+1^*r*_0_)∖*B*(*x*_0_,2^*j*^*r*_0_)_, *j* ∈ *N*∖{0}.

To finish the proof of Theorem 9, we only need to prove

(56) 1u(x0,r0)||{∑h|[bm,Sψ,a](fh)|r}1/rχB(x0,r0)||Lp(·)(Rn) ≤C||{∑h|fh|r}1/r||Mp(·),u(Rn).

Thus,

(57) 1u(x0,r0)||{∑h|[bm,Sψ,a](fh)|r}1/rχB(x0,r0)||Lp(·)(Rn) ≤Cu(x0,r0)||{∑h|[bm,Sψ,a](fh0)|r}1/rχB(x0,r0)||Lp(·)(Rn)  +Cu(x0,r0)∑j=1∞||{∑h|[bm,Sψ,a](fhj)|r}1/rχB(x0,r0)||Lp(·)(Rn) ≜E1+E2.

For the term *E*_1_, notice that supp *f*_*h*_^0^ ⊂ *B*(*x*_0_, 2*r*_0_); by Lemma 19 and inequality (32), we have

(58) E1≤Cu(x0,r0)||{∑h|fh0|r}1/r||Lp(·)(Rn)≤Cu(x0,2r0)u(x0,r0)1u(x0,2r0)||{∑h|fh|r}1/rχB(x0,2r0)||Lp(·)(Rn)≤C2nsup⁡y∈Rn,R>01u(y,R)||{∑h|fh|r}1/rχB(y,R)||Lp(·)(Rn)≤C||{∑h|fh|r}1/r||Mp(·),u(Rn).

Now we turn to estimate *E*_2_. According to the estimate of *S*_*ψ*,*a*_ in the proof of Theorem 6, we see that if *x* ∈ *B*(*x*_0_, *r*_0_), *z* ∈ *B*(*x*_0_, 2^*j*+1^ *r*_0_)∖*B*(*x*_0_, 2^*j*^ *r*_0_), *j* ≥ 1, then

(59) $$\begin{array}{c} S_{\psi ,a}\left(f_{h}^{j}\right)\left(x\right)\leq Ca^{3n/2+ɛ+\gamma }{\left(2^{j}r_{0}\right)}^{-n}{||f_{h}^{j}||}_{L^{1}(R^{n})}. \end{array}$$

Therefore,

(60) |[bm,Sψ,a](fhj)(x)| =|Sψ,a[(b(x)−b)mf](x)| ≤Ca3n/2+ɛ+γ(2jr0)−n||(b(·)−b)mfhj||L1(Rn).

Thus,

(61) E2 ≤Cu(x0,r0)  ×∑j=1∞||a3n/2+γ+ɛ(2jr0)−n{∑h||(b(·)−b)mfhj||L1(Rn)r}1/r     ×χB(x0,r0)||Lp(·)(Rn) ≤Cu(x0,r0)∑j=1∞(2jr0)−n  ×||||{∑h|(b(·)−b)mfhj|r}1/r||L1(Rn)χB(x0,r0)||Lp(·)(Rn) ≤Cu(x0,r0)∑j=1∞(2jr0)−n||(b−bB(x0,r0))mχB(x0,r0)||Lp(·)(Rn)  ×||{∑h|fhj|r}1/r||L1(Rn)  +Cu(x0,r0)∑j=1∞(2jr0)−n||χB(x0,r0)||Lp(·)(Rn)  ×||{∑h|(b−bB(x0,r0))mfhj|r}1/r||L1(Rn).

Using Hölder's inequality and Lemma 17, we get

(62) E2≤Cu(x0,r0)∑j=1∞(2jr0)−n||b||∗m||χB(x0,r0)||Lp(·)(Rn) ×||{∑h|fhj|r}1/r||Lp(·)(Rn)||χB(x0,2j+1r0)||Lp′(·)(Rn) +Cu(x0,r0)∑j=1∞(2jr0)−n||χB(x0,r0)||Lp(·)(Rn) ×||{∑h|fhj|r}1/r||Lp(·)(Rn) ×||(b−bB(x0,r0))mχB(x0,2j+1r0)||Lp′(·)(Rn)≤Cu(x0,r0)∑j=1∞(2jr0)−n[jm+1]||b||∗m ×||χB(x0,r0)||Lp(·)(Rn) ×||{∑h|fhj|r}1r||Lp(·)(Rn)||χB(x0,2j+1r0)||Lp′(·)(Rn).

And then, it follows from Lemma 16 and (22) that

(63) E2≤C||b||∗mCu(x0,r0)∑j=1∞(2jr0)−njm|B(x0,2j+1r0)| ×||χB(x0,r0)||Lp(·)(Rn)||χB(x0,2j+1r0)||Lp(·)(Rn)×u(x0,2j+1r0)1u(x0,2j+1r0) ×||{∑h|fhj|r}1/r||Lp(·)(Rn)≤C||b||∗mCu(x0,r0)∑j=1∞jm||χB(x0,r0)||Lp(·)(Rn)||χB(x0,2j+1r0)||Lp(·)(Rn) ×u(x0,2j+1r0) ×sup⁡y∈Rn,R>01u(y,R)||{∑h|fh|r}1/rχB(y,R)||Lp(·)(Rn)≤C||{∑h|fh|r}1/r||Mp(·),u(Rn).

Hence, Adding up the results of *E*_1_, *E*_2_, we have

(64) 1u(x0,r0)||{∑h|[bm,Sψ,a](fh)|r}1/rχB(x0,r0)||Lp(·)(Rn) ≤C||{∑h|fh|r}1/r||Mp(·),u(Rn).

The proof of Theorem 9 is accomplished.

**Proof of Theorem 10.**

For [*b* ^*m*^, *g*_*ψ*_], according to the estimate of *g*_*ψ*_ in the proof of Theorem 7, we see that if *x* ∈ *B*(*x*_0_, *r*_0_), supp *f*_*h*_^*j*^ ⊂ *B*(*x*_0_, 2^*j*+1^ *r*_0_)∖*B*(*x*_0_, 2^*j*^ *r*_0_), *j* ≥ 1, then

(65) $$\begin{array}{c} g_{\psi }\left(f_{h}^{j}\right)\left(x\right)\leq C{\left(2^{j}r_{0}\right)}^{-n}{||f_{h}^{j}||}_{L^{1}(R^{n})}. \end{array}$$

Thus,

(66) [bm,gψ]=|gψ[(b(x)−b)mf](x)|≤C(2jr0)−n||(b(·)−b)mfhj||L1(Rn).

Hence, similar to the proof of Theorem 9, and from inequality (2) in Lemma 19, it follows that

(67) ||{∑h|gψ(fh)|r}1/r||Mp(·),u(Rn) ≤C||{∑h|fh|r}1/r||Mp(·),u(Rn).

The proof of Theorem 10 is completed.

**Proof of Theorem 11.**

For [*b* ^*m*^, *g*_μ_*], according to the estimate of [*b* ^*m*^, *g*_μ_*] in the proof of Theorem 8, we get that if *x* ∈ *B*(*x*_0_, *r*_0_), supp *f*_*h*_^*j*^ ⊂ *B*(*x*_0_, 2^*j*+1^ *r*_0_)∖*B*(*x*_0_, 2^*j*^ *r*_0_), *j* ≥ 1, then

(68) $$\begin{array}{c} g_{\mu }^{*}\left(f_{h}^{j}\right)\left(x\right)\leq Ca^{3n/2+ɛ+\gamma }{\left(2^{j}r_{0}\right)}^{-n}{||f_{h}^{j}||}_{L^{1}(R^{n})}. \end{array}$$

Thus,

(69) [bm,gμ∗]=|gμ∗[(b(x)−b)mf](x)|≤Ca3n/2+ɛ+γ(2jr0)−n||(b(·)−b)mfhj||L1(Rn).

Hence, also similar to the proof of Theorem 9, it follows from inequality (3) in Lemma 19 that

(70) ||{∑h|[bm,gμ∗](fh)|r}1/r||Mp(·),u(Rn) ≤C||{∑h|fh|r}1/r||Mp(·),u(Rn).

The proof of Theorem 11 is accomplished.
